# Multi-strategic intervention to enhance implementation of healthy canteen policy: a randomised controlled trial

**DOI:** 10.1186/s13012-016-0537-9

**Published:** 2017-01-11

**Authors:** Luke Wolfenden, Nicole Nathan, Lisa M. Janssen, John Wiggers, Kathryn Reilly, Tessa Delaney, Christopher M. Williams, Colin Bell, Rebecca Wyse, Rachel Sutherland, Libby Campbell, Christophe Lecathelinais, Chris Oldmeadow, Megan Freund, Sze Lin Yoong

**Affiliations:** 1 Hunter New England Population Health, Locked Bag 10 Longworth Ave, Wallsend, New South Wales 2289 Australia; 2 The University of Newcastle, C/O Hunter New England Population Health, Locked Bag 10 Longworth Ave, Wallsend, New South Wales 2289 Australia; 3 Waurn Ponds Campus, School of Medicine, Deakin University, Geelong, 3220 Australia; 4 Hunter Medical Research Institute, 1 Kookaburra Circuit, New Lambton Heights, New South Wales 2305 Australia

**Keywords:** Healthy eating, Nutrition, Canteens, School, Policy, Children, Implementation

## Abstract

**Background:**

Internationally, governments have implemented school-based nutrition policies to restrict the availability of unhealthy foods from sale. The aim of the trial was to assess the effectiveness of a multi-strategic intervention to increase implementation of a state-wide healthy canteen policy. The impact of the intervention on the energy, total fat, and sodium of children’s canteen purchases and on schools’ canteen revenue was also assessed.

**Methods:**

Australian primary schools with a canteen were randomised to receive a 12–14-month, multi-strategic intervention or to a no intervention control group. The intervention sought to increase implementation of a state-wide healthy canteen policy which required schools to remove unhealthy items (classified as ‘red’ or ‘banned’) from regular sale and encouraged schools to ‘fill the menu’ with healthy items (classified as ‘green’). The intervention strategies included allocation of a support officer to assist with policy implementation, engagement of school principals and parent committees, consensus processes with canteen managers, training, provision of tools and resources, academic detailing, performance feedback, recognition and marketing initiatives. Data were collected at baseline (April to September, 2013) and at completion of the implementation period (November, 2014 to April, 2015).

**Results:**

Seventy schools participated in the trial. Relative to control, at follow-up, intervention schools were significantly more likely to have menus without ‘red’ or ‘banned’ items (RR = 21.11; 95% CI 3.30 to 147.28; *p* ≤ 0.01) and to have at least 50% of menu items classified as ‘green’ (RR = 3.06; 95% CI 1.64 to 5.68; *p* ≤ 0.01). At follow-up, student purchases from intervention school canteens were significantly lower in total fat (difference = −1.51 g; 95% CI −2.84 to −0.18; *p* = 0.028) compared to controls, but not in energy (difference = −132.32 kJ; 95% CI −280.99 to 16.34; *p* = 0.080) or sodium (difference = −46.81 mg; 95% CI −96.97 to 3.35; *p* = 0.067). Canteen revenue did not differ significantly between groups.

**Conclusion:**

Poor implementation of evidence-based school nutrition policies is a problem experienced by governments internationally, and one with significant implications for public health. The study makes an important contribution to the limited experimental evidence regarding strategies to improve implementation of school nutrition policies and suggests that, with multi-strategic support, implementation of healthy canteen policies can be achieved in most schools.

**Trial registration:**

Australian New Zealand Clinical Trials Registry (ACTRN12613000311752)

## Background

Nutrition policies governing the availability of foods in schools have been recommended by the World Health Organization to improve child nutrition [1]. In schools, such policies offer an opportunity to ensure that the foods made available to students comply with dietary guidelines. Systematic reviews of trials of the effectiveness of policies restricting the availability of unhealthy foods have consistently reported beneficial effects on child diet [2, 3]. As such, internationally, in jurisdictions where meals provided to children as part of school food services (e.g. UK), or where foods are available for sale to children at kiosks, tuckshops or canteens (e.g. Australia), countries have announced nutrition policies governing school food availability [1, 4–6].

Despite their popularity, research suggests that school nutrition polices are poorly implemented, limiting their potential public health impact. For example, in the USA, the Healthy, Hunger-Free Kids Act of 2010 (P.L. 111-296) required all schools participating in the National School Lunch Program to adopt and implement nutrition guidelines for all foods and beverages available at school (including competitive foods sold outside the school lunch programme) [7]. However, only 61% of students attend schools in a district with guidelines on competitive foods and less than 10% of middle and high school students were in a district that banned sugar sweetened beverages other than soda [7]. Similarly, in Australia, just 5–35% of schools in most states comply with government healthy canteen policies with marginal improvements in policy implementation occurring over time [8–10].

To maximise the public health benefits of school nutrition policies strategies are needed to overcome barriers to their implementation [11, 12]. Research investigating strategies to facilitate the implementation of health innovations in schools, however, is limited [13]. An updated Agency for Health Care Policy and Research systematic review identified just one randomised trial of a school-based strategy to improve the implementation of healthy eating policies or practices [14]. The trial reported little improvement in the provision of healthier school lunches between intervention schools who received training, technical assistance, resources and coalition building support to implement school food service guidelines, compared to control schools [15].

In the context of this limited evidence base, the primary aim of this study was to assess the effectiveness of a multi-strategic intervention to increase implementation of a state-wide healthy canteen policy (hereafter referred to as ﻿the ‘policy’). We also assessed the impact of policy implementation on the energy, total fat and sodium of children’s canteen purchases and on canteen revenue.

## Methods

The study was approved and the procedures monitored by the University of Newcastle Human Research Ethics Committee (Approval Number H-2008-0343) and the Hunter New England Human Research Ethics Committee (06/07/26/4.04) and was prospectively registered with Australian New Zealand Clinical Trials Register ACTRN12613000311752. Full details of the study methods are published [16]. The project was also approved by the NSW Department of Education and Communities (DEC) (#2012277).

### Context

In 2005, the New South Wales (NSW) Government launched a Healthy School Canteen Strategy (also known as *Fresh Tastes @ School*) to help prevent childhood obesity [4]. Consistent with the Australian Dietary Guidelines for Children and Adolescents, [17] the strategy classified foods sold by schools as ‘red’, ‘amber’ or ‘green’ based on their nutritional content. The strategy was adopted as policy by the government education department, and all government schools were mandated to remove items classified as ‘red’ from regular sale. Furthermore, schools were encouraged to ‘fill the menu’ with items classified as ‘green’ and ensure items classified as ‘amber’ did not dominate the menu. In 2007, the strategy was also supported by a ban on the sale of sugar sweetened drinks.

### Design and setting

The study employed a randomised trial design. Primary schools (those catering for children aged 5–12 years) in the Hunter region of NSW, Australia, with a canteen were randomised to receive a 12–14-month, multi-strategic implementation intervention or to a no intervention control group. To assess the primary trial outcome, data were collected at baseline (April to September, 2013) and at the completion of the implementation period (November, 2014 to April, 2015) (here after referred to as ‘follow-up’). Data to assess the nutritional quality of foods purchased at the school canteen were collected at follow-up only (March to April, 2015) on a nested sample of randomly selected schools.

### Participants and recruitment

Schools from the study region were randomly selected and invited to participate. To be eligible, schools were required to have an operational canteen and have either (i) one or more items on their canteen menu that was restricted for sale (‘red’ or ‘banned’) or (ii) less than 50% of menu items classified as healthy (‘green’ items). Non-government schools, schools with both primary and secondary students (i.e. central schools) and schools catering exclusively for children requiring specialist care were excluded. Recruitment continued until 70 schools provided consent for study participation. A nested sample of 50 participating schools was randomly selected, and consent was requested to perform observations to assess nutritional quality of canteen purchases.

### Random allocation and blinding

Following baseline data collection, schools were randomly allocated to the intervention or control using a permuted-block randomisation procedure of seven blocks to allow for equal numbers in each study arm. Schools were stratified for socioeconomic status of school locality using the Socio-Economic Index for Area (SEIFA 2006) [18] and randomised (1:1) using a random number function in Microsoft Excel. Data collectors were blind, but school staff (principals and canteen managers) were informed of their group allocation.

### Multi-strategic intervention group

The intervention sought to increase implementation of a healthy canteen policy. Specifically, the intervention assisted schools to remove ‘red’ and ‘banned’ canteen menu items from regular sale and increase the availability of ‘green’ menu items to more than 50% of all canteen menu items.

#### Conceptual model

The intervention was developed to address known barriers to the implementation of healthy canteen policies [11, 19, 20]. The selection of intervention components was guided by the Theoretical Domains Framework [21]. The scientific literature was reviewed, and workshops with canteen managers, teachers and health promotion practitioners with experience in working with school canteens were undertaken to identify barriers or facilitators to policy implementation. The research team then mapped potential behaviour change techniques (implementation strategies) to barriers identified in the literature reviews and through workshops [22] which were then refined based on considerations of feasibility, potential effectiveness and context. As a result, nine implementation strategies formed the intervention.

#### Implementation strategies

##### Policy implementation support

Schools were allocated a support officer with qualifications in nutrition and dietetics and experience in supporting schools to implement the policy. Support officers contacted canteen managers every 2 months (via email, telephone or in person) throughout the intervention and used a continuous quality improvement framework of repeated goal setting, action planning, self-monitoring and problem-solving with canteen managers.

##### Executive support

School principals were asked to communicate support for policy implementation and maintenance to teachers, parents, students and canteen managers during staff meetings, in newsletters and assemblies. Support officers also sought meetings with the executive of parent representative groups to garner their support for and input on policy implementation.

##### Consensus processes

Meetings between support officers and canteen staff were held to discuss and reach consensus regarding the policy, how best to implement it and to develop local canteen action plans to co-ordinate implementation tasks.

##### Training

Canteen managers, canteen staff and parent representatives were invited to attend a training workshop (5 h) with the aim of providing education and skill development in the policy, nutrition and food label reading, canteen stock and financial management, pricing and promotion, and change management. Training combined didactic and interactive components including opportunities for self-assessment, role play and facilitator provided feedback. Training was facilitated by a support officer. Schools were also offered a small reimbursement to cover the costs associated with canteen manager attendance at training.

##### Tools and resources

Canteen managers were provided with a ‘Canteen Resource Kit’ containing various printed and electronic instructional materials, including electronic menu and pricing templates, and a poster-sized checklist that prompted canteen managers to regularly review their canteen practices relating to Fresh Tastes @ School. Canteen managers also received kitchen equipment to the value of AUD$100.

##### Academic detailing

School canteen visits were conducted 1 and 3 months post-canteen manager training to enable support officers to observe the operational canteen environment, provide feedback and assist with problem-solving barriers to policy implementation.

##### Recognition

Schools with a menu assessed as adhering to the policy (i.e. greater than 50% ‘green’ items and no ‘red’ or ‘banned’ items) received a congratulatory letter and phone call from the research team and were publically acknowledged via marketing strategies.

##### Performance monitoring and feedback

Menu reviews were conducted quarterly (unless menus were unchanged), and the results were used to compile written feedback reports to the canteen manager and school principal. Verbal discussion of the reports occurred during academic detailing visits or via telephone support calls.

##### Marketing strategies

Quarterly project newsletters communicated key messages, provided information and case studies of successful implementation approaches to common barriers.

### Control group

No contact was made, and no resources provided to control schools during the intervention period by the research team.

### Data collection and measures

#### School and canteen characteristics

Canteen managers and school principals completed a scripted computer-assisted telephone interview (CATI) conducted by a trained research assistant at baseline and follow-up which assessed the number of students enrolled, canteen operational days, management structure and staffing profile.

#### Exposure to other nutrition interventions

During the follow-up CATI, principals and canteen managers were asked to report any exposure to and/or involvement in other initiatives to assist with the implementation of the policy.

#### Fidelity of intervention delivery

Project records were used to assess the delivery of each implementation strategy.

#### Perceived helpfulness of intervention support strategies

During the follow-up CATI, canteen managers of intervention schools were asked to rate, on a five-point Likert scale from ‘not helpful at all’ to ‘extremely helpful’, the following support strategies: resources, kitchen equipment, training workshop, email contact, menu audit and feedback report, newsletters, face-to-face meetings and telephone support calls.

#### Primary trial outcomes

The primary outcomes of the trial were (i) the proportion of schools with a canteen menu that did not contain foods or beverages restricted for sale (‘red’ and ‘banned’) under the policy and (ii) the proportion of schools where healthy canteen items (‘green items’) represented more than 50% of listed menu items.

At baseline and follow-up, copies of canteen menus were collected from all participating schools and audited by two dietitians independently. To ensure blinding to group allocation, all identifying information was removed from menus prior to auditing. Menu audits were conducted according to the protocol described by De Silva‐Sanigorski and colleagues (2011) [12]. Specifically, menu items were classified as ‘green’, ‘amber’ or ‘red’ (see Table 1) according to Fresh Tastes @ School criteria using food classification tools and guidelines published by the NSW Government. Information required to classify menu items that was not provided on the supplied canteen menus was subsequently collected from canteen managers via a telephone call by a research assistant. Discrepancies in product classification between dietitians were resolved through discussion between the two dietitians, or if agreement could not be reached, with a third independent dietitian.

**Table 1:** Examples of ‘green’, ‘amber’ and ‘red’ items based on “Fresh Tastes @ School”

Green	Amber	Red
Breads and breakfast cereals (those high in fibre and low in saturated fat and sugars). Fruits and vegetables. Reduced fat dairy products. Lean meat, fish and poultry. Water. 99% fruit juice in 200 mL serves or less.	Breakfast cereals (those refined with added sugar). Full fat dairy products. 99% fruit juice in serves greater than 200 mL but less than 300 mL. Fats, oils, spreads and gravies. Processed meats. Savoury commercial products, snack food bars and biscuits, savoury snack foods, muffins and cakes, ice creams and dairy desserts that fall below the Occasional Food Criteria [4]. Sugar sweetened drinks that have less than 300 kJ and/or less than 100 mg of sodium per serve.	All confectionery, deep fried foods and chocolate coated or premium ice creams. Foods that exceed the Occasional Food Criteria [4]. All sugar sweetened drinks with greater than 300 kJ per serve and/or greater than 100 mg of sodium per serve are banned from sale in school canteens.

#### Secondary trial outcomes

##### Nutritional quality of canteen purchases

Mean energy, total fat and sodium per student purchase were assessed via direct observations during one school day. Depending on the size of the canteen, and number of service lines, two or three research staff recorded all products sold, including those that were pre-ordered or sold over the counter, for each student purchase at both meal periods (‘recess and lunch’). In a sample of approximately 20% of schools, all purchases were independently recorded by two observers. Agreement between observers in the products recorded per student purchase was 95%. An inventory of the nutrient profile (including energy, total fat and sodium) of each item sold by canteens was recorded based on information provided on product labels, or for unpackaged foods, based on estimates from nutrient databases (Foodworks, version 7, Xyris Software, Highgate Hill, Australia) using ingredient lists and recipe information. The total energy (kJ), total fat (g) and sodium (mg) for each student purchase (consisting of one or more items) were calculated by combining the purchasing data with the nutrient profile of each item.

##### Canteen revenue

As a measure of potential adverse effects, canteen managers and/or principals were asked to provide a copy of the canteen’s financial records, reporting their yearly income and expenditure to enable calculation of canteen profitability. Additionally, during completion of the baseline and follow-up CATI, canteen managers were asked to report the approximate canteen revenue and profit (or loss) for the year preceding baseline data collection and the year of the intervention.

### Sample size and power

The sample was powered based on the primary trial outcomes. A sample of 70 schools (35 per group) was calculated to enable the study to detect as significant an absolute change in the primary trial outcomes of 35% with 80% power, assuming a control group prevalence of 15% at follow-up and an alpha of 0.05. Given a dose response relationships between fat [23] and sodium [24] intake and important health outcomes including precursors for chronic disease (such as blood pressure), sample size estimates for the secondary outcome of nutritional quality of canteen purchases were based on changes in energy intake between groups. Assuming 125 student canteen purchases occur daily per school, that a standard student lunch order contains 2100 kJ (sd = 1437) [25] and based on an ICC of 0.01, the participation of 40 schools (20 each arm) would enable detection of a 170-kJ difference between groups at follow-up with 80% power at the 0.05 significance level. At a population level, a reduction of this magnitude would be sufficient to avert anticipated increases in child body mass index [26].

### Analysis

All analyses were performed in SAS 9.3 (SAS Institute Inc., Cary, NC). Analyses of study outcomes were performed under an intention to treat framework. Between groups, differences in the primary outcome at follow-up were assessed using Fisher’s exact test and presented as relative risks (with approximate 95% confidence intervals). Analyses were first performed with all available data. Sensitivity analyses were then performed using multiple imputations to test the robustness of the findings to any bias introduced by missing data [27]. Subgroup analyses were performed to describe the impact of the intervention on primary trial outcomes by school size (number of children enrolled), where small was less than 160 students and medium/large was 160 students or greater, and the socio-economic region (derived from the SEIFA [18] indices assigned to the school postcode) in which the school is located by dichotomising these variables at the median. Linear regression models were used to assess between group differences in the mean energy, fat and sodium of student purchases at follow-up for all recess and lunch purchases, adjusting for potential clustering effect. Linear regression models were used to assess differential changes in mean canteen revenue between groups over time using all available data. In instances where canteen revenue could not be obtained from financial records (profit and loss statements), canteen manager self-report of revenue was used.

## Results

**Fig. 1:**
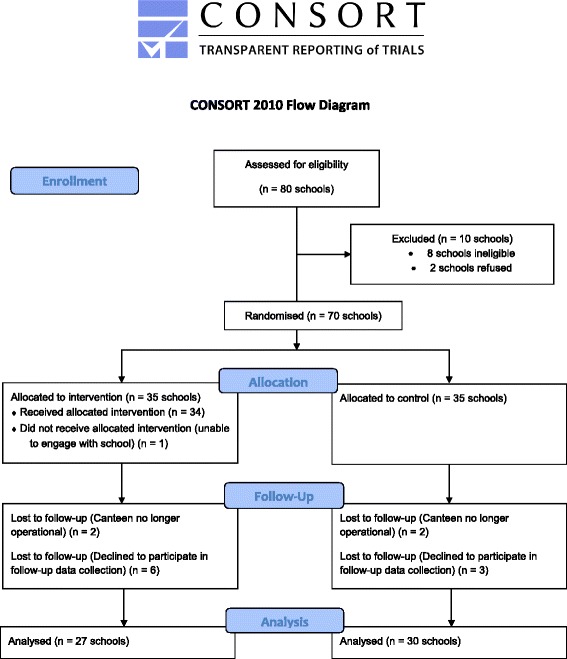
Consort 2010 flow diagram

Of the 124 schools randomly selected, 80 returned menus which were assessed for eligibility, eight were ineligible and two refused participation; 70 provided consent and were randomly allocated to the intervention or control group (Fig. 1). Of the schools who did not return menus, six had recently closed their canteen, 29 principals or school representatives could not be contacted, and nine schools refused to provide a menu.

The baseline characteristics of participating schools were similar between groups (Table 2). At baseline, all schools provided their menu for assessment and 69 principals and 69 canteen managers completed their respective telephone interview. At follow-up, 57 schools provided their menu for assessment and 56 principals and 49 canteen managers completed a telephone interview. There were no significant differences in baseline characteristic among schools that did and did not provide follow-up data (*p* = 0.19–0.54). Of the 50 schools randomised to participate in the observation of canteen purchases, 20 out of 24 intervention schools and 18 out of 26 controls provided consent. There were no significant differences among schools that did and did not consent to participate in observations in baseline school characteristics (*p* = 0.23–1.0).

**Table 2:** Baseline characteristics of participating schools by group

	Intervention	Control
	*n* = 35	*n* = 35
Mean (SD) number of students^a^	256 (147)	253 (173)^a^
Socioeconomic region (SEIFA 2006)		
Least advantaged	15 (42.9%)	16 (45.7%)
Most advantaged	20 (57.1%)	19 (54.3%)
Type of manager		
Paid manager	16 (45.7%)	16 (45.7%)
Volunteer manager	14 (40.0%)	15 (42.9%)
Other	5 (14.3%)	4 (11.4%)
Mean (SD) time as manager (in months)	51 (56)	57 (57)
Days of operation^b^		
5 days a week	15 (44.1%)	20 (57.1%)
3–4 days a week	14 (41.2%)	9 (25.7%)
1–2 days a week	5 (14.7%)	6 (17.1%)
Staffing of canteen		
All volunteer staff	19 (54.3%)	17 (48.6%)
Combination of volunteer and paid staff	15 (42.9%)	15 (42.9%)
Other	1 (2.9%)	3 (8.6%)

^a^Missing data from one control school

^b^Missing data from one intervention school

### Primary trial outcomes

As seen in Table 3, intervention schools were significantly more likely than control schools to have menus without ‘red’ or ‘banned’ items (RR = 21.11; 95% CI 3.30 to 147.28; *p* = <0.01), an effect that remained significant following sensitivity analyses and multiple imputation for missing data (RR = 12.80; 95% CI 1.81 to 90.61; *p* ≤ 0.01). Similarly, intervention schools were significantly more likely to have at least 50% of menu items classified as ‘green’ healthy foods than control schools (RR = 3.06; 95% CI 1.64 to 5.68; *p* ≤ 0.01). Sensitivity analyses support these findings (RR = 3.11; 95% CI 1.67 to 5.80; *p* ≤ 0.01). Overall, at follow-up, 58.7, 40.6 and 0.6% of canteen menu items from intervention schools were classified as ‘green’, ‘amber’ and ‘red’ respectively, compared with 43.7, 49.4 and 5.6%, respectively, among control schools. Intervention effects were significant across all subgroups, with the exception of small schools where there was no difference between groups in the proportion of schools where healthy canteen items (‘green’) represent >50% of products listed on the canteen menu (Table 4).

**Table 3:** Analysis of primary outcome variables

Variable	Baseline		Follow-up		Intervention vs control at follow-up	
	Int. (*n* = 35)	Cont. (*n* = 35)	Int. (*n* = 27)	Cont. (*n* = 30)	Relative risk (95% CI)	*P* value
Canteen menu does not contain foods and beverages restricted for sale (‘red’ or ‘banned’).	4 (11.43%)	6 (17.14%)	19 (70.37%)	1 (3.33%)	21.11 (3.03 to 147.28)	<0.01**
Healthy canteen items (‘green’) represent >50% of products listed on the canteen menu.	5 (14.29%)	7 (20.00%)	22 (81.48%)	8 (26.67%)	3.06 (1.64 to 5.68)	<0.01**

**p value less than 0.01

**Table 4:** Subgroup analysis of primary outcome variables

Variable	Baseline		Follow-up		Intervention vs control at follow-up	
	Int. (*n* = 35)	Cont. (*n* = 35)	Int. (*n* = 27)	Cont. (*n* = 30)	Relative risk (95% CI)	*P* value
Canteen menu does not contain foods and beverages restricted for sale (‘red’ or ‘banned’).						
School size						
•Small	3 (33.33%)	4 (36.36%)	7 (77.78%)	1 (14.29%)	5.44 (0.86 to 34.55)	0.04*
•Medium/large	1 (3.85%)	2 (8.70%)	12 (66.67%)	0	30.26 (1.91 to 478.45)	<0.01**
Socioeconomic region (SEIFA 2006)						
•Least advantaged	0	5 (31.25%)	9 (81.82%)	1 (7.69%)	10.64 (1.59 to 71.37)	<0.01**
•Most advantaged	4 (25.00%)	1 (5.26%)	10 (62.50%)	0	22.24 (1.41 to 350.79)	<0.01**
Healthy canteen items (‘green’) represent >50% of products listed on the canteen menu.						
School size						
•Small	0	1 (9.09%)	7 (77.78%)	3 (42.86%)	1.81 (0.72 to 4.57)	0.30
•Medium/large	5 (19.23%)	6 (26.09%)	15 (83.33%)	5 (22.73%)	3.67 (1.65 to 8.14)	<0.01**
Socioeconomic region (SEIFA 2006)						
•Least advantaged	2 (13.33%)	4 (25.00%)	11 (100.0%)	4 (30.77%)	3.25 (1.44 to 7.35)	<0.01**
•Most advantaged	3 (15.00%)	3 (15.79%)	11 (68.75%)	4 (23.53%)	2.92 (1.17 to 7.32)	0.01*

*p value less than 0.05; **p value less than 0.01

### Secondary outcomes

#### Nutritional quality of canteen purchases

At follow-up, student purchases from intervention schools were significantly lower in total fat (*p* = 0.03) compared to controls (Table 5).

**Table 5:** Mean (95% CI) energy, total fat and sodium of student purchases

Variable	Intervention	Control	Intervention vs control	
	(*n* = 2492)	(*n* = 2310)	Difference	*P* value
Energy (kJ)	801 (770 to 831)	933 (900 to 966)	−132.32 (−280.99 to 16.34)	0.08
Total fat (g)	5.83 (5.56 to 6.11)	7.34 (7.02 to 7.66)	−1.51 (−2.84 to −0.18)	0.03*
Sodium (mg)	261 (248 to 274)	308 (293 to 322)	−46.81 (−96.97 to 3.35)	0.07

*p value less than 0.05

#### Canteen revenue

Few schools provided data on canteen profit or loss. Analyses of available data found that changes in canteen profits over time between groups did not differ significantly (*p* = 0.34) (Table 6).

**Table 6:** Mean (SD) profits from available data

	Intervention	Control	*p* value
Baseline	*n* = 12^a^	*n* = 15^c^	0.34
	$6833.33 (5706.03)	$5920.00 (6459.23)	
Follow-up	*n* = 12^b^	*n* = 14^d^	
	$2678.83 (4121.86)	$4583.21 (4315.69)	

^a^Data missing for 25 schools. All data from self-report

^b^Data missing for 20 schools. All data from self-report

^c^Data missing for 15 schools. 11 from self-report and one from financial records

^d^Data missing for 16 schools. 11 from self-report and three from financial records

### Exposure to other nutrition interventions

Principals and/or canteen managers from 16 schools (seven interventions and nine controls) reported receiving additional support to implement a healthy canteen policy. Twenty-nine schools reported membership of the Healthy Kids Association (16 for intervention vs 13 for control), a government funded agency to support schools to implement healthy canteens, three of which (one intervention and two controls) reported that they had received support from the association during the study period. Two other sources of support most frequently reported by schools were Fresh for Kids, a multi-faceted programme run by Sydney Fruit Markets to promote the consumption of fresh fruit and vegetables and an active lifestyle amongst school-aged children (reported by five control schools), and Eat It To Beat It, a community-based programme run by the NSW Cancer Council that aims to increase fruit and vegetable consumption (reported by three intervention and one control school). Only two schools (one intervention and one control) reported Live Life Well @ School, a NSW Government-run programme to support schools to promote healthy eating and physical activity, as a source of additional canteen support.

### Fidelity of intervention delivery

Of intervention schools 94% (*n* = 33) received six or more of the nine intervention strategies. Attendance at the training workshop and the subsequent academic detailing visits was undertaken by 75% (*n* = 25) and 63% (*n* = 22) of schools, respectively. Recognition strategies were delivered to 57% (*n* = 20) of schools as not all canteen menus reached adherence to the policy during the implementation period. All other strategies were delivered to all but two intervention schools.

### Perceived helpfulness of intervention support strategies

Over 45% of canteen managers reported that each intervention component offered was considered very helpful (Table 7). The intervention component considered most helpful was ‘menu audit and feedback reports’.

**Table 7:** Helpfulness of intervention components

Intervention component	Not helpful	Slightly helpful–helpful	Very helpful–extremely helpful
Resource kit (*n* = 23)	0	8 (34.8%)	13 (56.5%)
Kitchen equipment (*n* = 24)	1 (4.2%)	4 (16.7%)	13 (54.2%)
Training workshop (*n* = 23)	0	4 (17.4%)	13 (56.5%)
Email contact (*n* = 23)	0	3 (13.0%)	15 (65.2%)
Menu audit and feedback report (*n* = 24)	0	4 (16.7%)	19 (79.2%)
Newsletters (*n* = 23)	2 (8.7%)	6 (26.1%)	11 (47.8%)
Face-to-face meetings (*n* = 23)	0	7 (30.4%)	14 (60.9%)
Telephone support calls (*n* = 23)	0	6 (26.1%)	14 (60.9%)

## Discussion

The failure of schools to implement government polices to reduce the availably of unhealthy foods at schools has been documented internationally [7–10] and undermines the potential public health benefits such policies were intended to deliver. To our knowledge, this is the first randomised trial designed specifically to test the impact of a strategy to improve implementation of a school nutrition policy [14]. The findings of this trial suggest that with multi-strategic support, implementation of healthy canteen policies can be achieved in the vast majority of schools (>70%).

The size of the intervention effect, equivalent to an absolute improvement within the intervention group of 54–67% on the primary trial outcomes, was larger than previous non-randomised trials of interventions which have sought to enhance implementation of nutrition policies or guidelines in school or childcare food service settings [15, 28]. The effect size was also larger than reported for strategies to support implementation of school-based health promotion programmes more broadly [29–34], which typically increase implementation by between 13 and 45%. Despite such encouraging findings, longer-term follow-up to examine whether improvements in policy implementation are sustained in the absence of intervention support is warranted.

The findings suggest that the intervention was successful in overcoming many of the barriers that reportedly hinder nutrition policy implementation in schools [11, 12]. However, the mechanism by which the intervention support strategies yielded improvements is unclear. A lack of understanding of the mechanism leading to implementation improvements has been previously identified as hindering optimization and the design of more efficient and effective implementation strategies [35, 36]. While the design of the implementation support was informed by considerable formative evaluation and utilised a comprehensive implementation theoretical framework, an examination of the impact of the strategy on hypothesised mediators was not conducted. The development and validation of tools to measure implementation constructs suitable for use in non-clinical settings would facilitate this research in the future [37].

Concerns regarding canteen profitability are frequently cited by canteen managers as an impediment to implementation of health canteen policies given the perceived higher costs, lower profit margin and greater labour required to prepare healthy foods for sale [11, 25, 38, 39]. Increases in the proportion of canteens making a loss have also been suggested in a study by Pettigrew and colleagues following the introduction of the Western Australian Healthy Food and Drink policy [40]. While statistical tests did not identify a significant reduction in profits of intervention canteens relative to control, the magnitude and direction of effect suggests that further investigation into the potential for such adverse effects is warranted.

The study findings should be considered in the context of the trial methods. The trial randomly sampled from the study region to maximise school representativeness and achieved relatively high participation rates. The generalisability of the findings to other jurisdictions or schools systems required to implement alternate school-based nutrition policies, however, is unknown. Risk of bias was reduced by central randomisation to groups, allocation concealment, blinded assessment and low trial attrition. Furthermore, project records suggest a high degree of intervention fidelity for a complex public health intervention with 94% of intervention schools receiving at least six of the nine intervention strategies. Student purchase data was included as a secondary trial outcome as additional resource became available to the researchers, in a subset of schools and at post-intervention only. Student level data were included in the trial register before any post-intervention data were collected but following initiation of the trial. While the inclusion of such data enables assessment of the impact of implementation of an evidence-based policy on the nutritional value of student purchases in the ‘real world’ the findings of the trial on this outcome should be considered in the context of these limitations. Missing data for assessments of canteen revenue, however, was considerable at both baseline and follow-up. As such, assessments of the impact of the intervention on canteen profits are likely to be unreliable. Finally, the trial included a relatively narrow range of potential implementation outcomes. Proctor and colleagues suggest that the inclusion of a broader range of implementation outcomes including acceptability, adoption, appropriateness, feasibility, penetration and sustainability may offer a more in-depth assessment of implementation processes and impact [41]. Consideration of such measures in future trials is therfore warranted.

## Conclusions

Without local level implementation support, government school nutrition policies are unlikely to yield improvements in public health nutrition. Notwithstanding the trial limitations, the study provides strong evidence supporting the effectiveness of one approach to support nutrition policy implementation in schools. While effective, providing multi-strategic implementation support to large numbers of schools may represent a considerable challenge for governments. Data regarding the perceived helpfulness of support strategies included in this trial, such as performance feedback, provide some suggestions as to which strategies may be most important. Nonetheless, future research to identify which intervention support strategies are the key drivers of improved policy implementation, and means of providing such support using lower cost delivery modalities (such as via telephone or the web) may be particularly beneficial.
